# Cardiometabolic and Cellular Adaptations to Multiple vs. Single Daily HIIT Sessions in Wistar Rats: Impact of Short-Term Detraining

**DOI:** 10.3390/metabo14080447

**Published:** 2024-08-14

**Authors:** Liliane Vanessa Costa-Pereira, Bruno Ferreira Mendes, Caíque Olegário Diniz Magalhães, Cíntia Maria Rodrigues, Júllia Alves de Andrade, Ramona Ramalho Souza de Pereira, Elizabethe Adriana Esteves, Ricardo Cardoso Cassilhas, Eric Francelino Andrade, Fernando Gripp, Flávio Castro de Magalhães, Kinulpe Honorato Sampaio, Alex Cleber Improta-Caria, Fabiano Trigueiro Amorim, Marco Fabrício Dias-Peixoto

**Affiliations:** 1 Multicenter Graduate Program in Physiological Sciences, Brazilian Society of Physiology, Diamantina 39100-000, MG, Brazil; lilianecostap@ufsj.edu.br (L.V.C.-P.); ferreira.mendes@ufvjm.edu.br (B.F.M.); caique.magalhaes@ufvjm.edu.br (C.O.D.M.); cintiamaria@usp.br (C.M.R.); elizabethe.esteves@ufvjm.edu.br (E.A.E.); ricardo.cassilhas@ufvjm.edu.br (R.C.C.); fernando.gripp@ufvjm.edu.br (F.G.); fcm@unm.edu (F.C.d.M.); kinulpe@ufvjm.edu.br (K.H.S.); 2 Graduate Program in Health Sciences, Federal University of the Jequitinhonha and Mucuri Valleys, Diamantina 39100-000, MG, Brazil; jullia.alves@ufvjm.edu.br (J.A.d.A.); ramona.souza@ufvjm.edu.br (R.R.S.d.P.); 3 Faculty of Health Sciences, Federal University of the Lavras, Lavras 37200-900, MG, Brazil; eric.andrade@ufla.br; 4 Laboratory of Biochemistry and Molecular Biology of the Exercise, University of Sao Paulo, São Paulo 05508-090, SP, Brazil; aleximprotacaria@usp.br; 5 Department of Health, Exercise and Sports Science, University of New Mexico, Albuquerque, NM 87131-0001, USA; amorim@unm.edu

**Keywords:** training cessation, accumulated exercise, sedentary behavior, exercise snacks

## Abstract

Multiple short daily bouts of HIIT are more effective than single daily sessions in improving cardiometabolic and cellular adaptations in rats. We hypothesize that a short period of detraining is sufficient to abolish the superior adaptive responses to multiple versus single daily sessions of HIIT in rats. Male rats were divided into untrained, 1xHIIT, and 3xHIIT groups. Over eight weeks, the 1xHIIT group performed 115 min single daily sessions of HIIT, while the 3xHIIT group performed three 5 min sessions with 4 h intervals. After training, both groups remained sedentary for four weeks (detraining). Resting oxygen consumption (VO2), body composition, glucose/insulin tolerance, and blood pressure were recorded. After euthanasia, cardiac function/histology and gastrocnemius mitochondrial density were analyzed. After training, both 1xHIIT and 3xHIIT protocols induced similar improvements in VO2, maximal oxygen uptake (VO2max), cardiac function/hypertrophy, and gastrocnemius mitochondrial density. These effects were maintained even after detraining. Only the 3xHIIT protocol improved insulin sensitivity. After detraining, this effect was abolished. After training, both 1xHIIT and 3xHIIT protocols reduced adiposity. After detraining, the adiposity increased in both groups, with a more pronounced increase in the 3xHIIT rats. A four-week detraining period abolishes the superior adaptive responses to multiple versus single daily HIIT sessions in rats.

## 1. Introduction

Detraining is defined as the partial or complete loss of adaptations to exercise as a result of training cessation [1]. Several factors, including the type, duration, and intensity of exercise, as well as the duration of training cessation, determine the magnitude of the loss of cellular and functional adaptations in response to detraining [2].

It is generally assumed that the complete loss of adaptations after the cessation of training occurs in approximately half the time it took to acquire those training adaptations [3]. Training cessation for specific periods throughout the year is a common practice in the general population; thus, understanding the consequences of training cessation on the cellular and functional adaptations obtained during the training period is of major importance.

One effective approach to enhancing cardiometabolic health involves interrupting prolonged sitting with brief exercise sessions throughout the day [4,5,6,7]. Recent studies have shown that accumulated exercise (several brief sessions of exercise during a day) induces greater adaptive responses in the cardiovascular and metabolic systems when compared with longer sessions of exercise once a day [8,9,10].

In the Costa-Pereira study [8], rats performed a 30–60 min moderate-intensity continuous training (MICT) protocol at 60–75% of the maximal workload. The difference between the accumulated MICT group and the traditional MICT group was that the accumulated MICT group performed three shorter daily sessions of 10–20 min with a 4 h interval between sessions, whereas the traditional MICT group performed the same volume and intensity of exercise with single daily sessions of 30–60 min. In the Mendes study [10], rats performed a 115 min HIIT protocol at 85–100% of maximal aerobic capacity either with single daily sessions of 15 min or three brief sessions of 5 min separated by an interval of 4 h between the sessions (accumulated HIIT). Notably, the superiority of training with multiple daily sessions over single daily sessions was more evident with the HIIT protocol compared to the MICT protocol. Specifically, the accumulated MICT and HIIT protocols were more effective than the traditional MICT and HIIT protocols, respectively, in reducing body and visceral adiposity. In addition, the accumulated HIIT was superior to the traditional HIIT in improving insulin sensitivity.

Here, we hypothesize that a short period of detraining (half the time of training) is sufficient to abolish the superior adaptive responses of the accumulated HIIT in rats. Therefore, this study was designed to investigate the effects of a detraining period of four weeks following eight weeks of a traditional versus an accumulated HIIT protocol on the adiposity, glucose tolerance, insulin sensitivity, cardiac function and hypertrophy, and skeletal mitochondrial density of Wistar rats.

## 2. Materials and Methods

### 2.1. Animals and Study Design

The experimental protocols were approved by the local ethical committee on animal use from the Federal University of Jequitinhonha and Mucuri Valleys (protocol #031/2016) and were conducted following the Guide for the Care and Use of Laboratory Animals published by the National Institute of Health (NIH Publication, 1996). Male Wistar rats, weighing between 220 and 260 g, were kept in separate cages for sixty days in a temperature-controlled room with 50% humidity, low noise, and standard laboratory food pellets (Nuvilab Nutrients LTDA, Colombo, PR, Brazil) and water ad libitum.

**Figure 1:**
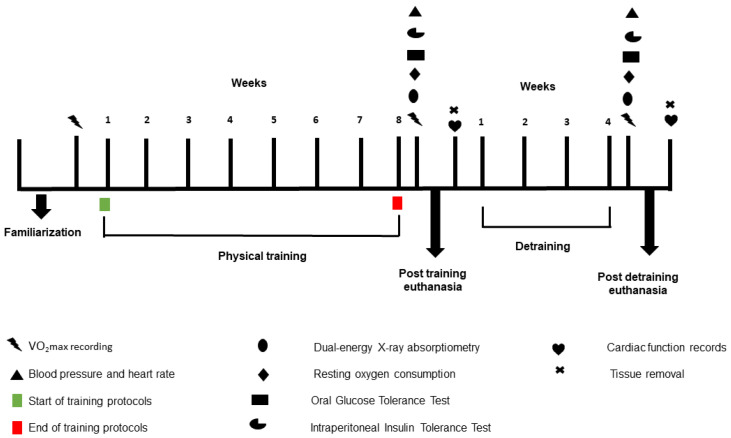
Study design.

Lights were turned on at 6 p.m. and turned off at 6 a.m. to ensure a 12:12 light–dark cycle for the animals. All experiments were conducted during the dark cycle. Seventy-two rats were randomly divided into three groups: Untrained (n = 24), HIIT performed with single daily sessions (1xHIIT, n = 24), and HIIT performed with three shorter daily sessions (3xHIIT, n = 24). The eight weeks of exercise training protocol were followed by an additional four weeks of detraining. Food intake and body weight were monitored weekly. A maximal oxygen consumption test (VO2max) was performed before exercise to determine exercise intensity and after the training and detraining periods. After the training and detraining protocols, glucose and insulin tolerance tests, blood pressure, heart rate, body composition, and resting oxygen consumption were measured. After the animals were euthanized, hearts, visceral fat, gastrocnemius, and hearts were harvested for heart function records and histological analyses. The study design is shown in Figure 1.

### 2.2. Test to Determine VO2max

The rats underwent a VO2max test as described by Hoydal et al. [11]. Briefly, the animals were acclimated to the exercise treadmill for five days by running at a low speed (10 m/min) for ten minutes daily. The VO2max test was conducted using a metabolic analyzer coupled to a metabolic chamber (Harvard Apparatus) 48 h after the last familiarization, 48 h before the first training session, 48 h after the last training session, and four weeks after the training cessation (detraining). A respiration-based software program (Metaoxy software, Harvard Apparatus, Madrid, Spain, version 2.2.01) was used to record the calorimetric parameters. The treadmill running speed was increased by 3 m/min every 2 min until the VO2max speed, which was obtained when oxygen consumption (VO2) reached a plateau despite the increase in running speed. The maximal speed (Vmax) for the exercise training prescription was determined to be the treadmill speed at VO2max.

### 2.3. Training and Detraining Protocols

**Figure 2:**
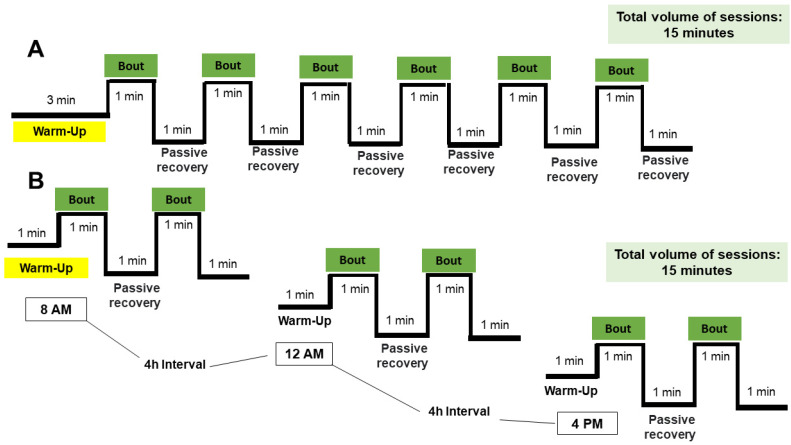
Exercise training protocols. 1xHIIT: High-Intensity Interval Training performed with single daily sessions (**A**); 3xHIIT: High-Intensity Interval Training performed with three shorter daily sessions (**B**).

Figure 2 presents both exercise protocols design. The exercise training protocols started 4 h after the VO2max protocol. The untrained rats were placed in the same room as the HIIT groups carried out the exercise protocols. The untrained rats also exercised on the treadmill for ten minutes each week at 10 m/min. The 1xHIIT and 3xHIIT groups exercised five days per week for eight weeks. The 15 min sessions were performed once a day by the 1xHIIT rats. They consisted of a 3 min warm-up, and six 1 min bouts separated by a 1 min passive recovery period (Figure 2A). The 3xHIIT rats also underwent daily 115 min sessions, which were divided into three shorter sessions of a 1 min warm-up, 1 min exercise bouts separated by 1 min passive recovery (a total of 5 min), and a 4 h break between sessions (Figure 2B). The volume of exercise was identical for both HIIT groups over the eight weeks of training and the exercise intensity was progressively increased: 85% Vmax (1st week), 90% Vmax (2nd and 3rd weeks), 95% Vmax (4th and 5th weeks), and 100% Vmax (6th, 7th, and 8th weeks). At the end of the fourth week of training, a second VO2max test was performed to determine a new maximal running speed (Vmax). To avoid any potential circadian influence on training adaptation, the 1xHIIT group was split into three subgroups that began the training sessions at the same times as the 3xHIIT group (8:00 a.m., 12:00 p.m., and 4:00 p.m.). To evaluate the detraining effects, the rats were kept in their cages from the 8th week to the 12th week. 

### 2.4. Body Composition Analysis

Dual-energy X-ray absorptiometry (iDXA, G.E. Healthcare, Chicago, IL, USA) was used to measure body composition 24 h following the exercise session and four weeks after training cessation. Animals were placed in the scanning area of the apparatus and given intraperitoneal injections of xylazine (8–15 mg/kg) and ketamine (60–80 mg/kg). The animals were positioned so that the center of anatomical points like the pelvis, head, spine, and hind legs were beneath the delineated sagittal line. Lean mass and body fat percentage were determined using commercial analysis software (enCORE, G.E. Healthcare, Chicago, IL, USA) [12].

### 2.5. Heart Rate and Systolic Blood Pressure Records

Heart rate and tail systolic blood pressure were measured 48 h after the training program and after the four-week detraining period using noninvasive tail-cuff plethysmography (Costa Pereira et al. [8]).

### 2.6. Resting Oxygen Consumption Records

Seventy-two hours after the last exercise session and four weeks after detraining, resting oxygen consumption was assessed. After an 8 h fast, the rats were put in a metabolic chamber with an airflow of 1.0 L/min, which was connected to an Oxyleptro computer-monitored indirect calorimeter (Harvard Apparatus, Barcelona, Spain, version 2.2.01) for the evaluation of resting oxygen consumption. A respiration-based software program (software Metaoxy, Harvard Apparatus, Spain) was used to measure calorimetric parameters [13].

### 2.7. Oral Glucose Tolerance Test (OGTT)

Immediately following the resting oxygen consumption records, the OGTT was conducted. A gavage was used to deliver dextrose (2 g/kg of body weight at 50% solution) and, by a tiny puncture in the rat tail just before (0 min) and then again 30, 60, and 120 min after the gavage, blood glucose levels were measured using an ACCU-CHEK (Advantage Glucose Analyzer, Roche Diagnostics Corporation, Indianapolis, IN, USA) [14].

### 2.8. Intraperitoneal Insulin Tolerance Test (IpITT)

The animals were given an intraperitoneal dose of insulin (1 I.U./kg B.W.) 48 h after the OGTT, following an eight-hour fast. Blood glucose levels were ascertained using the identical OGTT methodology described above. To account for potential variations in baseline glucose values between groups, all data were normalized and expressed as a percentage of baseline glucose concentration. The glucose measurements (% baseline glucose × 60 min) were used to compute the area under the curve (AUC) [14].

### 2.9. Euthanasia

Half of the animals in each group were euthanized after the training period, and the other half were euthanized after the four-week withdrawal period. Following an intraperitoneal injection of 400 I.U. heparin, the animals were decapitated and their visceral fat, heart, and gastrocnemius muscle were removed and weighed. Hearts were perfused using the Langendorff technique to measure cardiac function. Sections of the retroperitoneal fat were taken out, weighed, and fixed in 4% Bouin to estimate the area of the adipocytes. A segment of the gastrocnemius muscle was processed (by transmission electron microscopy) for assessments of mitochondrial density [13,14].

### 2.10. Heart Function Records

The heart was dissected and perfused with Krebs-Ringer solution in the Langendorff apparatus (ML785B2, ADInstruments, Dunedin, New Zealand), at 37 ± 1 °C, constant pressure (65 mm Hg), with oxygenation (5% CO_2_ and 95% O_2_), KH2PO4 (1.17 mmol/L), MgSO_4_ (1.17 mmol/L), CaCl_2_ (2.50 mmol/L), glucose (11.65 mmol/L), and NaHCO_3_ (26.30 mmol/L). Heart rate (H.R.) and the contractility (+dP/dt) and relaxation (−dP/dt) indexes were recorded using the AcqKnowledge program (TSD 104A, Biopac Systems Inc., Santa Barbara, CA, USA) [14]. The left ventricles were removed, weighed, and separated into fragments for histological analyses after cardiac function records.

### 2.11. Histological Analyses

The fragments of retroperitoneal fat and gastrocnemius muscles were fixed in 10% formalin for 48 h. Subsequently, they were dehydrated using increasing alcohol gradients (70%, 80%, 90%, and 100%) and then infiltrated and included in paraffin. The samples were assembled into blocks and cut into 5 mm sections with intervals of 20 cuts using a microtome. The sections were stained with hematoxylin and eosin for analysis. Cross-sections were used to evaluate the cell area length of at least 100 adipocytes or myocytes per animal (~10 fields per animal) [13].

### 2.12. Mitochondrial Density Analyses

Three animals in each group had dissected their gastrocnemius (posterior midbelly segments). After an overnight period at 4 °C, the specimens were fixed in Karnovsky’s solution (2.5% glutaraldehyde and 2% paraformaldehyde) in 0.1 M cacodylate buffer pH 7.4. After that, they were postfixed for two hours in a solution containing 1.5% (*w*/*v*) potassium ferrocyanide and 2% (*w*/*v*) osmium tetroxide to improve the contrast of the organelles. After an overnight soak in 2% uranyl acetate, the specimens were cleaned in distilled water. The samples were embedded in Epon 812 after undergoing successive dehydration in baths of varying grades of ethanol. The specimens were stained with Reynolds lead citrate after being sectioned into 50 nm sections. Using an FEI Tecnai G2—12 Spirit at 80 kV, point resolution was determined by transmission electron microscopy (TEM). The resolution of the obtained photos was 1373 × 1070 pixels. For every animal, twenty-five electron micrographs were captured at a magnification of ×11,000. Images from the core regions of muscle fibers were chosen at random, and ImageJ was used for analysis. Using the standard point approach, the volume densities (Vv) of both altered and normal mitochondria were calculated by projecting a 700 × 700 nm grid, or 130 points, onto each picture. According to a prior description [15], altered mitochondria were classified as those that had a bloated appearance, a rarefied matrix, and damaged cristae.

### 2.13. Statistical Analysis

The data are presented as mean ± standard deviation. The normality of the data was assessed using the Shapiro–Wilk test. All analyses were conducted in a blinded manner. One-way (main factor = 3 groups) or two-way (3 groups vs. pre, post-training, and detraining) analysis of variance was used, followed by Tukey’s post hoc test (Statistica software, v8.0, StatSoft, Inc., Tulsa, OK, USA). Statistical significance was set at *p* ≤ 0.05.

## 3. Results

### 3.1. Training and Detraining Effects of 1xHIIT and 3xHIIT on VO2max, Body Mass and Food Intake

**Figure 3:**
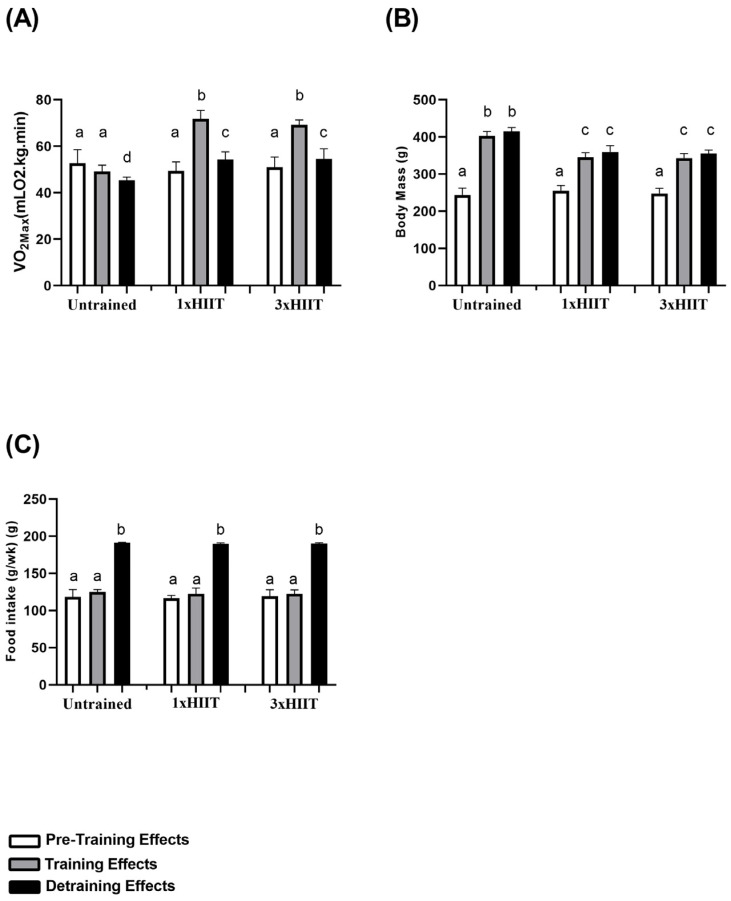
Training and detraining effects of single versus three shorter daily sessions of HIIT on (**A**) VO2max, (**B**) body mass, and (**C**) food intake. Data are presented as mean ± S.D. n = 24/group. Two-way ANOVA followed by Tukey test. Untrained, non-exercised group; 1xHIIT, High-Intensity Interval Training performed in single daily sessions; 3xHIIT, High-Intensity Interval Training performed in three shorter daily sessions. Different letters indicate statistical differences.

Figure 3 presents the VO2max (A), body mass (B), and food intake (C) data of the untrained, 1xHIIT, and 3xHIIT groups before and after the training and detraining periods. Before training, VO2max, body mass, and food intake were similar among groups (*p* > 0.05). After training, VO2max increased in the 1xHIIIT and 3xHIIT rats (1xHIIT: *p* < 0.0001 and 3xHIIT: *p* < 0.0001) and did not change in the Untrained group, confirming the effectiveness of the training protocols. After four weeks of training cessation, VO2max decreased in all groups (Untrained: *p* < 0.05; 1xHIIT: *p* < 0.0001 and 3xHIIT: *p* < 0.0001). However, the VO2max of both trained groups remained significantly higher than the pre-training values (1xHIIT: *p* < 0.0001 and 3xHIIT: *p* < 0.0001). No differences in the VO2max were found between the 1xHIIT and 3xHIIT rats after detraining (1xHIIT vs. 3xHIIT: *p* > 0.05).

After the training period, body mass increased in all groups (Untrained: *p* < 0.0001; 1xHIIT: *p* < 0.0001 and 3xHIIT: *p* < 0.0001). However, the increase in body mass was lower in the trained groups compared to that of the Untrained group (1xHIIT vs. Untrained: *p* < 0.0001; 3xHIIT vs. Untrained: *p* < 0.0001), and the 1xHIIIT and 3xHIIT rats showed similar increases in body mass (1xHIIT vs. 3xHIIT: *p* > 0.05). After four weeks of training cessation, the body mass of all groups did not differ from their post-training values (Untrained: *p* > 0.05; 1xHIIT: *p* > 0.05 and 3xHIIT: *p* > 0.05), and the body mass of trained rats remained significantly lower than that of the Untrained rats (1xHIIT vs. Untrained: *p* < 0.0001; 3xHIIT vs. Untrained: *p* < 0.0001). Food intake did not change after eight weeks of training protocols in all groups (Untrained: *p* > 0.05; 1xHIIT: *p* > 0.05 and 3xHIIT: *p* > 0.05). However, food intake significantly increased in all groups after four weeks of training cessation (Untrained: *p* < 0.0001; 1xHIIT: *p* < 0.0001 and 3xHIIT: *p* < 0.0001) and did not differ among groups (1xHIIT vs. Untrained: *p* > 0.05; 3xHIIT vs. Untrained: *p* > 0.05; 1xHIIT vs. 3xHIIT: *p* > 0.05).

### 3.2. Training and Detraining Effects of 1xHIIT and 3xHIIT on Body Composition

**Figure 4:**
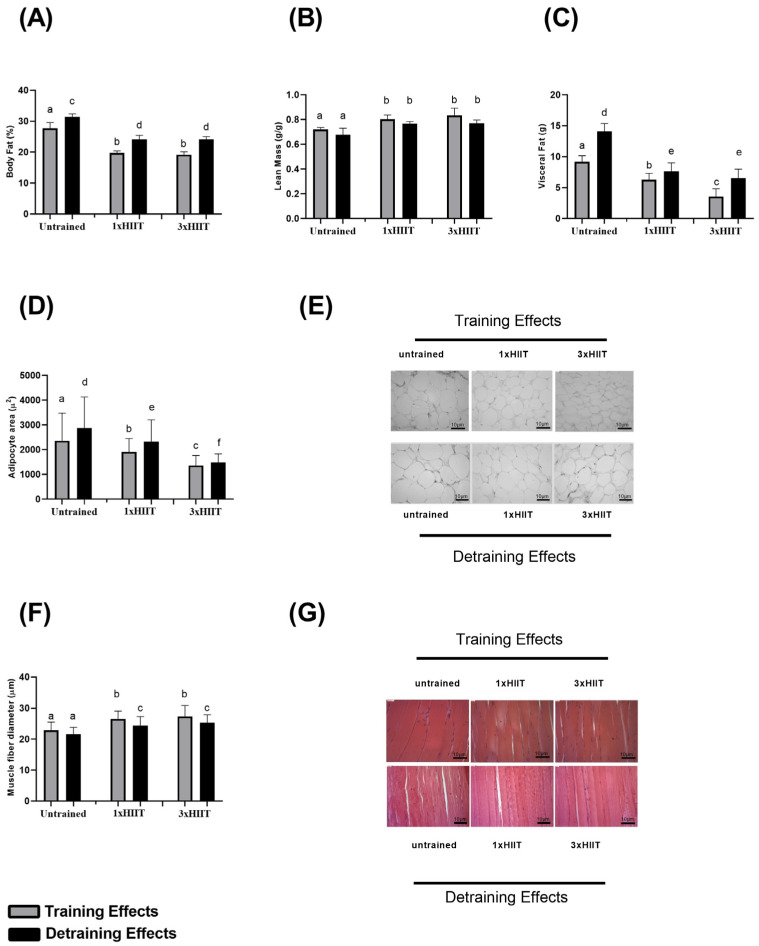
Training and detraining effects of single versus three shorter daily sessions of HIIT on body composition, adipocyte size, and muscle fiber diameter (gastrocnemius muscle). Body fat percentage (%) ((**A**), n = 24); lean mass (*g*/*g*) ((**B**), n = 24); visceral fat percentage (%) ((**C**), n = 24); adipocyte size (µ2) ((**D**), n = 60 cells/group from four independent experiments); representative H&E slides of adipocytes in the retroperitoneal fat (**E**); muscle fiber diameter gastrocnemius, n = 38 fibers/group from four independent experiments (**F**); representative H&E slides of gastrocnemius muscle (**G**). Two-way ANOVA followed by Tukey test. Untrained, non-exercised group; 1xHIIT, High-Intensity Interval Training performed in single daily sessions; 3xHIIT, High-Intensity Interval Training performed in three shorter daily sessions. Different letters indicate statistical differences.

Figure 4 presents the data on body fat mass (A), lean mass (B), visceral fat mass (C), adipocyte size (D–E), and muscle fiber diameter (F–G) during the training and detraining periods. After training, the HIIT groups exhibited lower body fat mass than the untrained rats (1xHIIT vs. Untrained: *p* < 0.0001; 3xHIIT vs. Untrained: *p* < 0.0001). No differences in body fat mass were found between the 1x and 3xHIIT groups (1xHIIT vs. 3xHIIT: *p* > 0.05). After four weeks of detraining, body fat mass increased in all groups. However, it remained lower in the HIIT groups compared to the untrained rats (1xHIIT vs. Untrained: *p* < 0.0001; 3xHIIT vs. Untrained: *p* < 0.0001). Body fat mass did not differ between the 1x and 3xHIIT rats after detraining (*p* > 0.05). 

After training, the lean mass of the 1xHIIT and 3xHIIT groups was higher than that of the Untrained rats (1xHIIT vs. Untrained: *p* < 0.01; 3xHIIT vs. Untrained: *p* < 0.0001), and it was similar between the HIIT groups (1xHIIT vs. 3xHIIT: *p* > 0.05). After the detraining period, there was no change in lean mass across all groups (Untrained: *p* > 0.05; 1xHIIT: *p* > 0.05; 3xHIIT: *p* > 0.05), and it remained higher in the HIIT groups compared to the Untrained group (1xHIIT vs. Untrained: *p* = 0.0014; 3xHIIT vs. Untrained: *p* = 0.0006).

After training, both HIIT groups exhibited lower visceral fat mass (1xHIIT vs. Untrained: *p* < 0.0001; 3xHIIT vs. Untrained: *p* < 0.0001) and adipocyte size (1xHIIT vs. Untrained: *p* < 0.0001; 3xHIIT vs. Untrained: *p* < 0.0001) compared to untrained rats. Furthermore, compared to the 1xHIIT rats, the 3xHIIT rats presented lower visceral fat mass (3xHIIT vs. 1xHIIT: *p* < 0.0001) and adipocyte size (3xHIIT vs. 1xHIIT: *p* < 0.0001).

After detraining, both visceral fat mass and adipocyte size increased in all groups (visceral fat mass—Untrained: *p* < 0.0001; 1xHIIT: *p* < 0.0001; 3xHIIT: *p* < 0.0001; adipocyte size—Untrained: *p* < 0.0001; 1xHIIT: *p* < 0.0001; 3xHIIT: *p* < 0.0001); however, they were still significantly lower in the HIIT groups compared to untrained rats (visceral fat mass—1xHIIT vs. Untrained: *p* < 0.0001; 3xHIIT vs. Untrained: *p* < 0.0001; 1xHIIT vs. 3xHIIT: *p* = 0.3078). Notably, after the detraining period, the 3xHIIT group exhibited similar visceral fat mass to the 1xHIIT group (1xHIIT vs. 3xHIIT: *p* > 0.05), but the adipocyte size remained lower in the 3xHIIT rats compared to the 1xHIIT rats (1xHIIT vs. 3xHIIT: *p* < 0.0001).

After training, both HIIT groups presented larger skeletal muscle fiber diameters compared to the Untrained group (1xHIIT vs. Untrained: *p* < 0.0001; 3xHIIT vs. Untrained: *p* < 0.0001; 1xHIIT vs. 3xHIIT: *p* > 0.05). After four weeks of detraining, the muscle fiber diameter remained larger in both HIIT groups compared to the Untrained group, and it remained similar between the HIIT groups (1xHIIT vs. Untrained: *p* = 0.0029; 3xHIIT vs. Untrained: *p* < 0.0001; 1xHIIT vs. 3xHIIT: *p* > 0.05).

### 3.3. Training and Detraining Effects of 1xHIIT and 3xHIIT on Resting Oxygen Consumption and Blood Glucose Homeostasis

**Figure 5:**
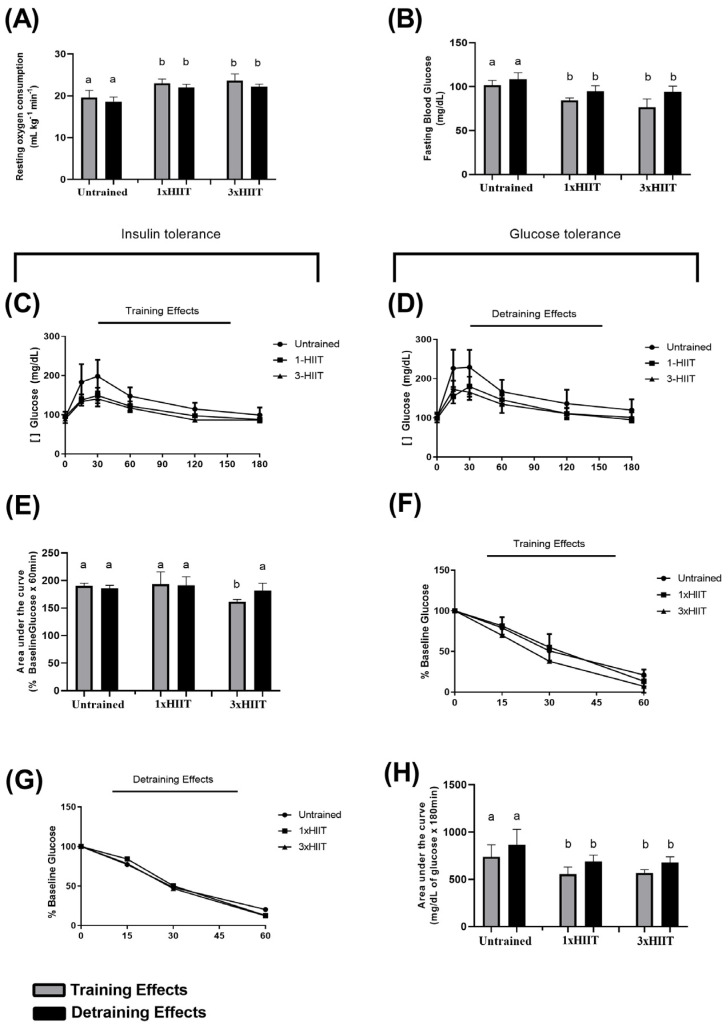
Training and detraining effects of single versus three shorter daily sessions of HIIT on (**A**) resting oxygen consumption, (**B**) fasting blood glucose, (**C**–**E**) glucose tolerance, and (**F**–**H**) insulin tolerance. Data are presented as mean ± S.D. n = 24/group. Two-way ANOVA followed by Tukey test. Untrained, non-exercised Group; 1xHIIT, High-Intensity Interval Training performed in single daily sessions; 3xHIIT, High-Intensity Interval Training performed in three shorter daily sessions. Different letters indicate statistical differences.

Figure 5 shows the results of resting oxygen consumption (A), fasting blood glucose (B), glucose tolerance (C–E), and insulin tolerance (F–H) tests after training and detraining. 

After the training period, both HIIT groups exhibited similarly higher resting oxygen consumption (1xHIIT vs. Untrained: *p* < 0.0001; 3xHIIT vs. Untrained: *p* < 0.0001; 1xHIIT vs. 3xHIIT: *p* = 0.7775) and lower fasting blood glucose (1xHIIT vs. Untrained: *p* < 0.0001; 3xHIIT vs. Untrained: *p* < 0.0001; 1xHIIT vs. 3xHIIT: *p* = 0.0468) compared to the Untrained group. These results remained unaltered after four weeks of detraining for both resting oxygen consumption (Untrained: *p* > 0.05; 1xHIIT: *p* > 0.05; 3xHIIT: *p* > 0.05; 1xHIIT vs. Untrained: *p* < 0.0001; 3xHIIT vs. Untrained: *p* < 0.0001) and fasting blood glucose levels (Untrained: *p* > 0.05; 1xHIIT: *p* > 0.05; 3xHIIT: *p* > 0.05; 1xHIIT vs. Untrained: *p* < 0.0001; 3xHIIT vs. Untrained: *p* < 0.0001).

After training, both 1x and 3xHIIT groups had similar improvements on the glucose tolerance test (1xHIIT vs. Untrained: *p* = 0.0049; 3xHIIT vs. Untrained: *p* = 0.0098; 1xHIIT vs. 3xHIIT: *p* = 0.9998).

After the four weeks of detraining, the improvement in glucose tolerance was preserved in both the 1x and 3xHIIT groups (1xHIIT vs. Untrained: *p* = 0.0059; 3xHIIT vs. Untrained: *p* = 0.0033; 1xHIIT vs. 3xHIIT: *p* = 0.9878). Similar to our findings in the previous study, only the 3xHIIT protocol improved insulin tolerance after training (1xHIIT vs. Untrained: *p* > 0.9999; 3xHIIT vs. Untrained: *p* = 0.0048; 1xHIIT vs. 3xHIIT: *p* = 0.0013). However, this positive effect from the 3xHIIT protocol was abolished after detraining (1xHIIT vs. Untrained: *p* > 0.05; 3xHIIT vs. Untrained: *p* > 0.05; 1xHIIT vs. 3xHIIT: *p* > 0.05).

### 3.4. Training and Detraining Effects of 1xHIIT and 3xHIIT on Cardiovascular Parameters

**Figure 6:**
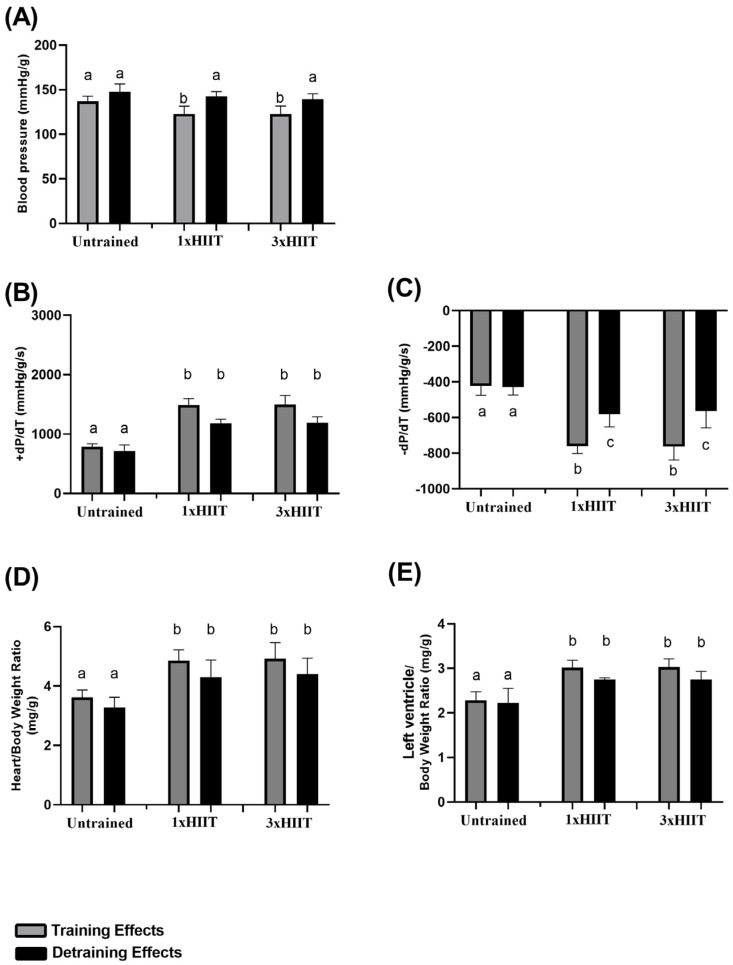
Training and detraining effects of single versus three shorter daily sessions of HIIT on cardiovascular parameters. (**A**) Blood pressure; (**B**) +dP/Dt; (**C**) -dP/Dt; (**D**) heart/body weight ratio; (**E**) left ventricle/body weight ratio. Data are presented as mean ± S.D. n = 24/group. Two-way ANOVA followed by Tukey test. Untrained, non-exercised Group; 1xHIIT, High-Intensity Interval Training performed in single daily sessions; 3xHIIT, High-Intensity Interval Training performed in three shorter daily sessions. Different letters indicate statistical differences.

Figure 6 shows the data of blood pressure (A), +dP/Dt (B), −dP/Dt (C), heart/body weight (D), and left ventricle/body weight ratios (E) after training and detraining with 1x and 3xHIIT.

After eight weeks of training, both HIIT groups exhibited lower blood pressure compared to the Untrained group (1xHIIT vs. Untrained: *p* = 0.0212; 3xHIIT vs. Untrained: *p* = 0.0179; 1xHIIT vs. 3xHIIT: *p* > 0.05). After the detraining period, the blood pressure of both HIIT groups increased to the levels observed in the untrained animals (1xHIIT vs. Untrained: *p* > 0.05; 3xHIIT vs. Untrained: *p* = 0.8968; 1xHIIT vs. 3xHIIT: *p* > 0.05).

After training, the HIIT groups had higher cardiac contractility ( + dP/Dt: 1xHIIT vs. Untrained: *p* < 0.0001; 3xHIIT vs. Untrained: *p* < 0.0001; 1xHIIT vs. 3xHIIT: *p* > 0.05) and cardiac relaxation (-dP/Dt: 1xHIIT vs. Untrained: *p* < 0.0001; 3xHIIT vs. Untrained: *p* < 0.0001; 1xHIIT vs. 3xHIIT: *p* > 0.05) compared to the untrained rats. After detraining, cardiac contractility remained similar to that observed after training in all groups (1xHIIT vs. Untrained: *p* < 0.0001; 3xHIIT vs. Untrained: *p* < 0.0001; 1xHIIT vs. 3xHIIT: *p* > 0.05). Cardiac relaxation did not change in the untrained rats (*p* > 0.05) and decreased similarly in both HIIT groups (1xHIIT: *p* < 0.0001; 3xHIIT: *p* < 0.0001; 1xHIIT vs. 3xHIIT: *p* > 0.05) after the detraining period; however, cardiac relaxation remained higher in both HIIT groups compared to the untrained rats (1xHIIT vs. Untrained: *p* < 0.0001; 3xHIIT vs. Untrained: *p* < 0.0001).

After training, both HIIT protocols induced similar cardiac hypertrophy as demonstrated by the heart/body weight (1xHIIT vs. Untrained: *p* < 0.0001; 3xHIIT vs. Untrained: *p* < 0.0001; 1xHIIT vs. 3xHIIT: *p* > 0.05) and left ventricle/body weight (1xHIIT vs. Untrained: *p* < 0.0001; 3xHIIT vs. Untrained: *p* < 0.0001; 1xHIIT vs. 3xHIIT: *p* > 0.05) ratios. 

The increase in heart/body weight (1xHIIT: *p* > 0.05; 3xHIIT: *p* > 0.05; 1xHIIT vs. 3xHIIT: *p* > 0.05) and left ventricle/body weight ratios (1xHIIT: *p* > 0.05; 3xHIIT: *p* > 0.05; 1xHIIT vs. 3xHIIT: *p* > 0.05) induced by training was maintained after four weeks of training cessation. No differences were observed in cardiac hypertrophy indexes in the untrained rats after the training and detraining periods (*p* > 0.05).

### 3.5. Training and Detraining Effects of 1xHIIT and 3xHIIT on Mitochondrial Densities of Cardiac and Skeletal Muscles

**Figure 7:**
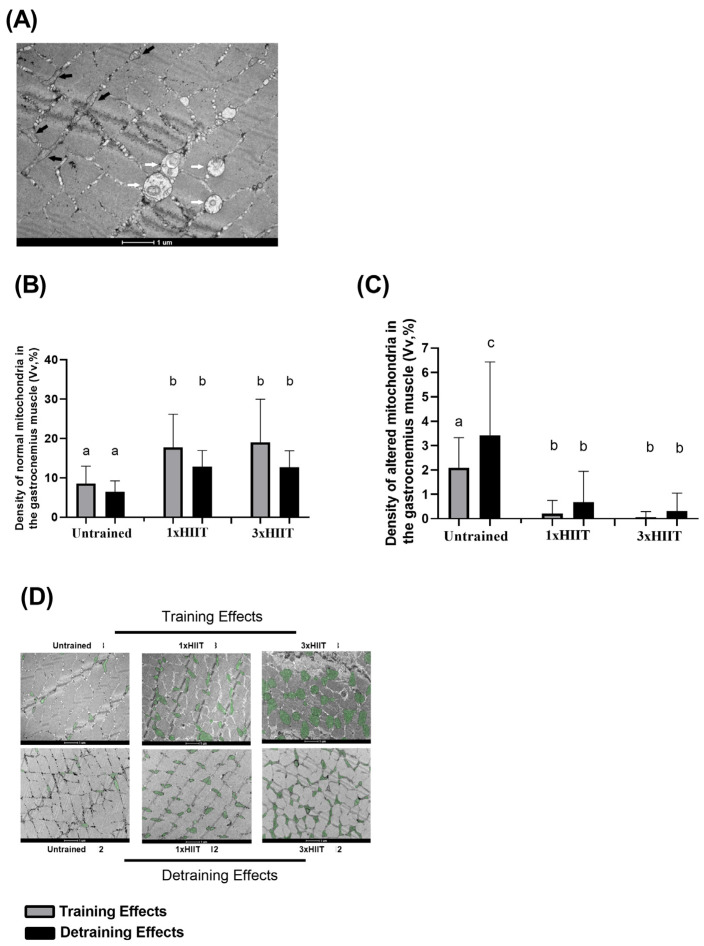
Training and detraining effects of single versus three shorter daily sessions of HIIT on mitochondria density. Representative mitochondria of the gastrocnemius muscle for Untrained, 1xHIIT, and 3xHIIT groups (**A**). White arrows show normal mitochondria, and black arrows show altered mitochondria. Quantification of normal (**B**) and altered (**C**) mitochondria density in gastrocnemius muscle fibers. Transmission electron micrographs of transverse sections of the gastrocnemius muscle (**D**). Data are presented as mean ± S.D. Two-way ANOVA followed by Tukey test. Untrained, non-exercised group; 1xHIIT, High-Intensity Interval Training performed in single daily sessions; 3xHIIT, High-Intensity Interval Training performed in three shorter daily sessions. Different letters indicate statistical differences.

The training and detraining effects of 1xHIIT and 3xHIIT on the density of normal and altered mitochondria in the gastrocnemius muscle are shown in Figure 7A–D. Figure 7A shows a representative cross-section of the gastrocnemius muscle by transmission electron microscopy, with white arrows indicating normal mitochondria and black arrows indicating altered mitochondria. After training, both HIIT groups had a similar increase in the density of normal mitochondria (1xHIIT vs. Untrained: *p* < 0.0001; 3xHIIT vs. Untrained: *p* < 0.0001; 1xHIIT vs. 3xHIIT: *p* > 0.05). These results were not altered by the four-week detraining period (1xHIIT vs. Untrained: *p* < 0.0001; 3xHIIT vs. Untrained: *p* < 0.0001; 1xHIIT vs. 3xHIIT: *p* > 0.05). After training, the density of altered mitochondria was similarly lower in the HIIT groups compared to the untrained group (1xHIIT vs. Untrained: *p* < 0.0001; 3xHIIT vs. Untrained: *p* < 0.0001; 1xHIIT vs. 3xHIIT: *p* > 0.05). After detraining, untrained rats showed an increase in the density of altered mitochondria (*p* > 0.05), and the density of altered mitochondria in the HIIT groups was similar to that observed after the training period (1xHIIT vs. Untrained: *p* = 0.0446; 3xHIIT vs. Untrained: *p* = 0.0001; 1xHIIT vs. 3xHIIT: *p* > 0.05).

## 4. Discussion

This study aimed to investigate the effects of four weeks of training cessation after an HIIT protocol performed with single daily sessions versus three shorter daily sessions in rats. Overall, although certain functional and cellular adaptations acquired after eight weeks of training were maintained, other adaptations were partially or completely lost after the detraining period. Moreover, there were both similar and distinct detraining effects between the 1x and 3x HIIT protocols. The main findings are discussed below.

### 4.1. Cardiovascular System

Exercise training is widely recognized for its beneficial effects on the cardiovascular system [8,16]. Physiological cardiac hypertrophy induced by training is associated with an enhancement in cardiac contractility and relaxation [17]. Our previous studies demonstrated that both MICT [8] and HIIT [10], when performed over eight weeks with three shorter daily sessions, induced cardiac hypertrophy and improvements in heart contractility and relaxation, similar to those observed with single daily exercise sessions. In this study, we observed that the cardiac hypertrophy and improvements in cardiac function induced by eight weeks of 1x and 3x HIIT were maintained even after four weeks of training cessation. Olah et al. [16] investigated the effects of detraining on cardiac function and hypertrophy in rats. The rats were trained for 12 weeks, and after eight weeks of detraining, they had a complete reversal of cardiac hypertrophy and improvements in cardiac contractility (+dP/dt) and relaxation (−dP/dt) indexes induced by training. 

The reversal of hypertrophy and cardiac function after detraining observed by Olah et al. [18] differs from the results of our current study. We found that the 1x and 3x HIIT rats maintained cardiac hypertrophy and improvement in cardiac function induced by training even after four weeks of training cessation. The differences between our results and those of Olah may be primarily explained especially by the differences between our detraining protocol and the one used by Olah et al. While we used a four-week detraining protocol, Olah used eight weeks. Thus, it is plausible to speculate that if the detraining period in the current study were extended from 4 to 8 weeks, the 1x and 3x HIIT groups in the current study might have a complete reversal of the training-induced cardiac adaptations.

Studies have shown that the effects of training on blood pressure reduction are less pronounced in normotensive rats compared to hypertensive rats. Additionally, the blood pressure reduction induced by training is abolished in normotensive rats after a short period of detraining [19,20,21]. These studies showed that hypertensive rats had a significant reduction in blood pressure after 8–10 weeks of endurance training, and this effect was maintained even after 1–5 weeks of detraining [19,20,21]. However, in normotensive rats, the training-induced a small reduction in blood pressure [19,20] or no alteration [21]. After the short detraining period, blood pressure in normotensive rats returned to pre-training levels. These studies suggest that the greater reduction in blood pressure and the maintenance of this training adaptation after a short period of detraining only in hypertensive rats may be related to the more pronounced beneficial effects of training on reducing sympathetic activity and improving vascular function in these animals. Thus, we believe that if the 1x and 3xHIIT rats in the current study were hypertensive, a greater reduction in blood pressure would be observed after training. Furthermore, in contrast to the findings in the normotensive rats in this study, this training adaptation would likely be maintained after the short period of detraining.

### 4.2. Metabolic System

Human and animal studies have consistently demonstrated a decrease in VO2max following a period of detraining ([22,23,24] Gripp et al.). The decline in VO2max following detraining can be primarily attributed to the reversal of the cardiovascular and metabolic adaptations that develop in response to aerobic exercise training. A decline in cardiac function [23], a reduction in muscle mass [24], and a decrease in the activity of mitochondrial enzymes responsible for aerobic energy synthesis [25] are major contributors to the decline in VO2max after a period of detraining. Nevertheless, the VO2max of both the 1x and 3x HIIT rats did not return to their pre-training values.

The maintenance of some central and peripheral training adaptations in the 1x and 3x HIIT groups after the detraining period may partly explain the partial maintenance of the VO2 max in both groups following the detraining period. Specifically, the increase in cardiac contractility and hypertrophy, the increase in the density of normal mitochondria, and the decrease in the density of altered mitochondria in the skeletal muscle found after eight weeks of training were maintained even after 4 weeks of detraining. 

Previous studies have demonstrated the superior effects of multiple shorter bouts of intense exercise on insulin sensitivity in rats [10] and humans [26,27] compared to single daily sessions of exercise. Here, we confirmed that the 1x and 3xHIIT protocols enhance insulin sensitivity with superior effects for the 3xHIIT protocol. However, after detraining both 1x and 3xHIIT rats lost this training adaptation and the insulin tolerance of the 3xHIIT group was similar to that observed in the 1xHIIT rats. Thus, the superior effect of the 3xHIIT protocol in enhancing insulin tolerance was abolished after detraining. 

The faster reduction in insulin sensitivity after four weeks of detraining in animals previously trained with three shorter daily sessions of HIIT compared to those trained with longer, single daily sessions of HIIT may be attributed to the more abrupt cessation of daily exercise sessions in the 3xHIIT group. Specifically, while detraining resulted in the cessation of training involving one daily exercise session in the 1xHIIT group, it led to the cessation of training involving three daily exercise sessions in the 3xHIIT group. Previous studies have proposed that multiple, shorter bouts of intense exercise result in repeated, vigorous muscle contractions over the course of a day [10,27]. This may lead to higher muscle glucose uptake and insulin sensitivity compared to single daily sessions of exercise. The faster decline of insulin sensitivity after a period of detraining in the 3xHIIT rats of this study may be related to the loss of frequent muscle contractions previously maintained by regular and multiple daily bouts of exercise.

### 4.3. Body Composition

We showed that after four weeks of detraining, both HIIT groups exhibited increased body fat, visceral fat, and adipocyte size. However, the adiposity of the HIIT groups remained lower than that observed in the untrained group. This increase in fat mass after a detraining period is supported by other authors [17,23]. The rise in adiposity in the 1x and 3x HIIT animals probably resulted from a reduction in energy expenditure and a significant increase in food consumption following exercise cessation. However, the adiposity of the HIIT groups was still lower than that of the untrained group after the four weeks of training cessation. Since the food intake of the HIIT groups was similar to that of the untrained rats during the four weeks of detraining, the lower adiposity of the HIIT rats, even after four weeks of detraining, may be explained by the basal oxygen consumption results. In particular, the basal oxygen consumption of the HIIT animals increased significantly after training and did not decrease after the detraining period. Another interesting point is that the increase in visceral fat and adipocyte size after the detraining period was more pronounced in the 3x HIIT group than in the 1x HIIT group. 

Immediately after the eight weeks of training, visceral fat mass and adipocyte size were lower in the 3x HIIT animals than in the 1x HIIT animals. Nevertheless, after four weeks of detraining, both visceral fat mass and adipocyte size were similar between the HIIT groups. Both HIIT groups exhibited similar food intake and basal metabolic rates after the training and detraining periods. Thus, these variables cannot explain the greater visceral fat gain during the detraining period in the 3x HIIT group compared to the 1x HIIT group. In our previous study, we hypothesized that the total energy expenditure of shorter multiple exercise bouts throughout a day could surpass that of single daily sessions. 

Multiple daily exercise bouts may induce multiple episodes of excess post-exercise oxygen consumption (EPOC), thereby leading to higher energy expenditure than that of single daily sessions [5]. If our hypothesis is correct, it could explain the faster increase in body observed in the 3x HIIT animals compared to the 1x HIIT animals after four weeks of detraining. If accumulated exercise results in greater energy expenditure and a more effective reduction in body fat compared to single-session exercise, it is reasonable to speculate that the cessation of accumulated exercise could have a more pronounced impact in reducing the daily energy expenditure, resulting in a faster increase in body fat.

### 4.4. Study Limitations and Future Directions

A complete characterization of the mechanisms involved with the detraining effects after the 1x and 3xHIIT protocols is beyond the scope of this study. Moreover, it is imperative to conduct human studies to confirm the detraining effects in response to single versus multiple shorter daily sessions of HIIT.

### 4.5. Practical Implications

In the real world, understanding the training and detraining effects of multiple daily HIIT sessions has practical implications for many adults. The accumulated HIIT protocol can be easily incorporated into daily routines, especially for those with busy schedules or sedentary occupations. Multiple shorter exercise sessions can help break up long periods of sedentary behavior, improve exercise adherence, and reduce the risk of chronic disease. This strategy can be a practical and effective public health intervention that promotes sustained physical activity and better overall health outcomes.

## 5. Conclusions

We revealed that the superiority of cardiometabolic and cellular adaptations in response to multiple daily HIIT sessions compared to single daily HIIT sessions is lost after a short period of detraining. However, most of the adaptation losses after detraining in the 1x and 3xHIIT rats were only partial. Thus, both training modalities may be interesting choices for individuals who experience regular training interruptions over the course of a year.

## Data Availability

The data supporting the findings of this study are available within the article. The data are not publicly available due to privacy restrictions. Further inquiries can be directed to the corresponding authors.
